# Replacing middle colic artery arising from the splenic artery—an arterial variety in a patient undergoing total pancreatoduodenectomy

**DOI:** 10.1093/jscr/rjae609

**Published:** 2024-09-23

**Authors:** Robert Karitnig, Christian Margreiter, Doris Wagner, Valerie Fanny Wienerroither, Andri Lederer, Hans Michael Hau, Peter Kornprat, Emina Talakic, Robert Sucher

**Affiliations:** Department of General-, Visceral- and Transplant Surgery, Medical University of Graz, Auenbruggerplatz 29, 8036 Graz, Austria; Department of General-, Visceral- and Transplant Surgery, Medical University of Graz, Auenbruggerplatz 29, 8036 Graz, Austria; Department of General-, Visceral- and Transplant Surgery, Medical University of Graz, Auenbruggerplatz 29, 8036 Graz, Austria; Department of General-, Visceral- and Transplant Surgery, Medical University of Graz, Auenbruggerplatz 29, 8036 Graz, Austria; Department of General-, Visceral- and Transplant Surgery, Medical University of Graz, Auenbruggerplatz 29, 8036 Graz, Austria; Department of General-, Visceral- and Transplant Surgery, Medical University of Graz, Auenbruggerplatz 29, 8036 Graz, Austria; Department of General-, Visceral- and Transplant Surgery, Medical University of Graz, Auenbruggerplatz 29, 8036 Graz, Austria; Division of General Radiology, Department of Radiology, Medical University of Graz, Auenbruggerplatz 9, 8036 Graz, Austria; Department of General-, Visceral- and Transplant Surgery, Medical University of Graz, Auenbruggerplatz 29, 8036 Graz, Austria

**Keywords:** arterial variation, coeliac trunk, HBP surgery, pancreatic surgery

## Abstract

Knowledge of variations in arterial vascular supply is crucial in HPB and general surgery. Although the arterial configuration of the coeliac trunk and the superior mesenteric artery had been investigated, there are still arterial branching patterns to be described. We herein present the case of an 84-year-old male patient who underwent total pancreatectomy due to a not specified pancreas head tumor with a replacing right hepatic artery according to Michel’s classification III and a replacing middle colic artery arising from the splenic artery and running on the ventral side of the pancreas. To the best of our knowledge, this arterial branching pattern has never been described so far. In this case, two arterial variations had been presented with a type III arterial supply according to Michel’s classification, and a replacing middle colic artery arising from the SA.

## Introduction

Knowledge of variations in arterial vascular supply is crucial in HPB and general surgery. Visualization, including 3D-reconstruction, has significantly improved over the last few years [1]. Therefore, unforeseen findings during surgery can be reduced.

The most frequent anatomy of the upper gastrointestinal arteries is present only in ~55% to 70% of all individuals [2, 3]. Coeliac trunk (CTr) divides into the splenic artery (SA), the left gastric artery (LGA), and the common hepatic artery (CHA). CHA gives rise to the gastroduodenal artery (GDA), the right gastric artery (RGA), and continues as the proper hepatic artery (PHA). The superior mesenteric artery (SMA) supplies the small intestine, the ascending colon, and the transverse colon up to the left colic flexure and communicates with the branches of the inferior mesenteric artery described as Drumond’s and Riolan’s anastomosi. Especially the arterial anatomy of the liver is variable, as categorized by Michels [4].

Although the arterial configuration of the CTr and the SMA had been investigated, there are still arterial branching patterns to be described. This case report describes an arterial variation of a middle colic artery (MCA) arising from the SA.

## Case report

We herein present the case of an 84-year-old male patient who underwent total pancreatectomy due to a not specified pancreas head tumor. During weekly interdisciplinary case discussion, the preoperative CT scans of this patient were discussed, showing a replacing right hepatic artery in Michel’s classification III. The 3D-reconstruction showed an arterial vessel arising from the SA, running on the ventral side of the pancreas, heading into the mesocolon (Fig. 2). The tumor was initially classified as borderline respectable due to contact of the tumor with the portal vein of 180°.

**Figure 1:**
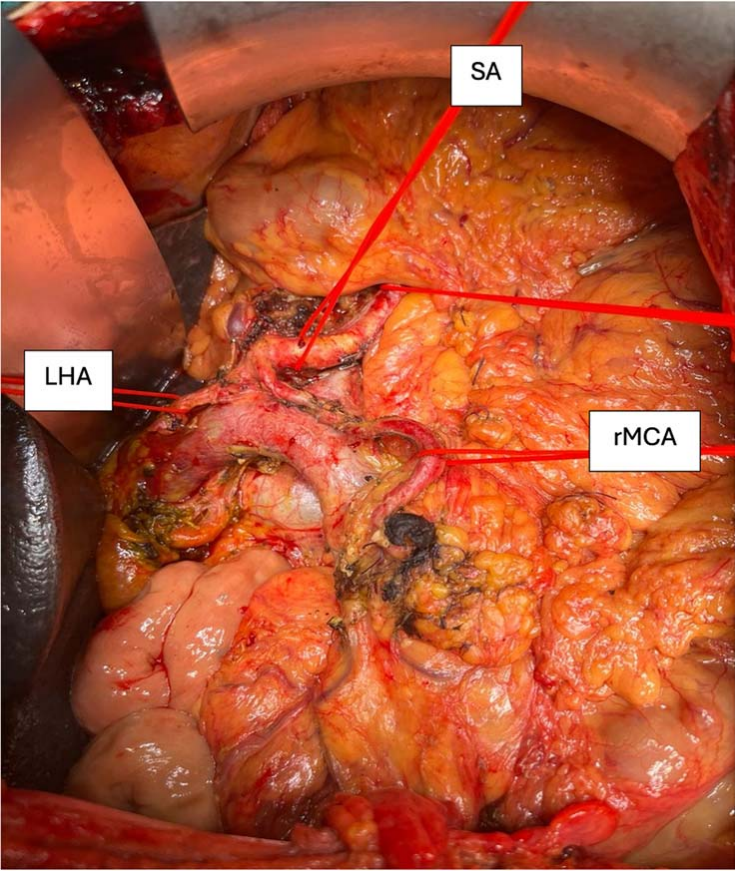
Image of the post-resection situs showing the coeliac trunk with its branches marked with vessel loops. SA, splenic artery; LHA, left hepatic artery; rMCA, replacing middle colic artery.

An open surgical approach using a median laparotomy was performed. After preparation of the replacing RHA arising from the SMA, the MCA arising from the SA was isolated, running across the ventral side of the pancreas and heading into the transverse mesocolon. The clamping test of the vessel showed ischemia in the transverse colon. The vessel was followed to its origin to the SA. The distal course was followed to check any connection to the SMA. No arterial blood supply was found to arise from the SMA, as shown in Fig. 1. The aberrant artery must be considered as replacing MCA. During the whole procedure, this artery was secured, the resection was successfully completed, and a total pancreatectomy was performed.

**Figure 2:**
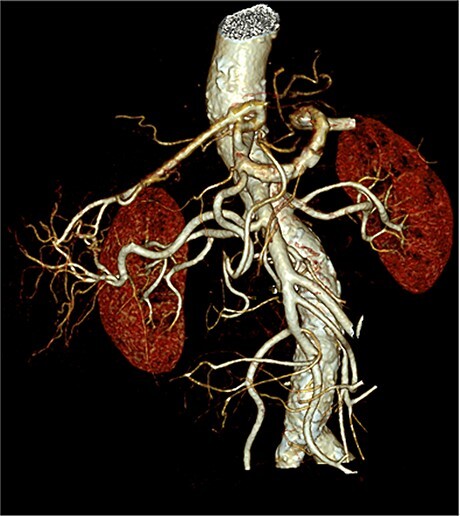
3D-reconstruction of the arterial branching pattern of the whole coeliac trunk and the superior mesenteric artery.

## Discussion

To the best of our knowledge, this arterial branching pattern has never been described so far, neither in a human body nor in another vertebrate. A similar case of an MCA arising from the SA has been described by Pretterklieber and Pretterklieber [5] with the difference that an aberrant artery dorsal to the pancreas was present supplying the transverse mesocolon. Yano *et al.* [6] investigated the MCA in a total of 143 patients retrospectively and found two cases (1.4%) of an MCA arising from the CTr. The anatomical relationship between the artery and the pancreas has not been mentioned in the publication.

Not only the arteries supplying the liver are highly variable, much more the whole arterial supply of the abdomen must be considered as variable. In this case, two arterial variations had been presented with a type III arterial supply according to Michel’s classification, and a replacing MCA arising from the SA.
